# Active site determinants of yeast Pah1 phosphatidate phosphatase activity and cellular functions

**DOI:** 10.1016/j.jbc.2025.110492

**Published:** 2025-07-17

**Authors:** Geordan J. Stukey, Parth K. Sharma, Ruta Jog, Joanna M. Kwiatek, Gil-Soo Han, George M. Carman

**Affiliations:** Department of Food Science and the Rutgers Center for Lipid Research, Rutgers University, New Brunswick, New Jersey, USA

**Keywords:** phospholipid, phosphatidate, diacylglycerol, triacylglycerol, PA phosphatase, lipin, lipid metabolism

## Abstract

In the yeast *Saccharomyces cerevisiae*, the *PAH1*-encoded phosphatidate (PA) phosphatase plays a major role in the control of diacylglycerol and PA, which are crucial for the synthesis of the storage lipid triacylglycerol and membrane phospholipids as well as for diverse cellular processes. The catalytic core of Pah1 contains the haloacid dehalogenase–like domain, a catalytic domain in diverse phosphatases with the conserved motifs I–IV. In this work, we found the four active site motifs in Pah1 by sequence alignment and AlphaFold modeling and identified Arg-445 as an additional residue conserved in motif II of Pah1 and its orthologs. Mutational analyses of the Pah1 active site motifs showed that the conserved residues (Asp-398 and Asp-400 in motif I, Thr-443 and Arg-445 in motif II, Lys-496 in motif III, and Gly-529, Asn-530, and Asp-534 in motif IV) are essential for PA phosphatase (PAP) activity and the related cellular functions of the enzyme. The limited proteolysis analysis of unphosphorylated Pah1, which mimics the functional dephosphorylated form of Pah1, indicates that its overall structure is not affected by the active site mutations. In the liposome-binding assay, the active site mutations in Pah1 did not affect its association with the membrane. These findings demonstrate that the active site motifs are essential for Pah1 PAP activity and provide a mechanistic basis for lipid-associated diseases caused by mutations in the human lipin PAP.

In the yeast *Saccharomyces cerevisiae*, Pah1 catalyzes the dephosphorylation of phosphatidate (PA) to diacylglycerol (DAG) (1) (Fig. 1). The Mg^2+^-dependent PA phosphatase (PAP) plays a crucial role in lipid metabolism by regulating the cellular levels of PA and DAG (1), which are key intermediates in the synthesis of membrane phospholipids and the storage lipid triacylglycerol (TAG), respectively (Fig. 1) (2, 3, 4, 5). The substrate PA is activated with CTP to form CDP–DAG, which is used for the synthesis of major membrane phospholipids phosphatidylserine (PS), phosphatidylethanolamine (PE), phosphatidylcholine (PC), phosphatidylinositol (PI), phosphatidylglycerol, and cardiolipin (2, 3, 5) (Fig. 1). The product DAG is a direct precursor of TAG whose synthesis is increased in the stationary phase (6, 7) (Fig. 1). DAG can also be channeled into the synthesis of PC and PE in yeast cells supplemented with choline and ethanolamine, respectively, which is essential for the mutants lacking the phospholipid synthesis *via* CDP–DAG (Fig. 1) (1, 5, 8).

**Figure 1:**
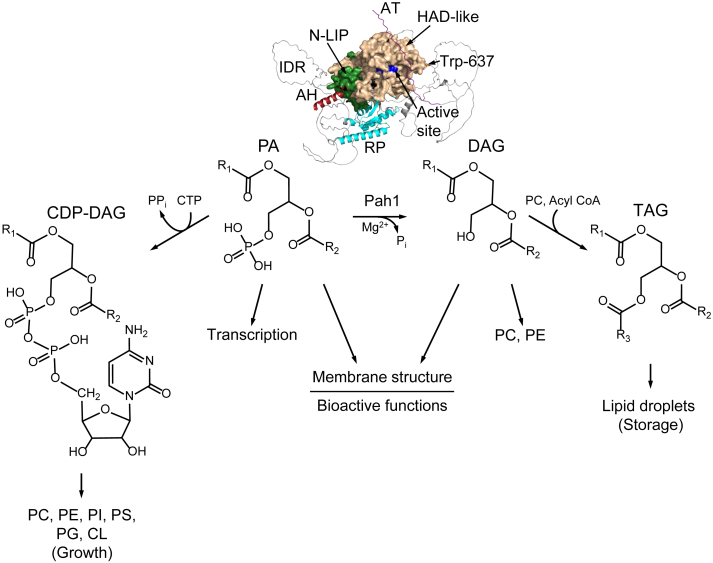
**PAP reaction catalyzed by Pah1 and its role in lipid synthesis and cell physiology.** The structures of Pah1 and its substrate (PA) and product (DAG), and their derivatives (CDP–DAG and TAG) are shown. The Pah1 structure predicted by AlphaFold (88, 89, 90) is visualized by the PyMol program. The domains/regions of Pah1 include the amphipathic helix (AH); N-LIP domain; haloacid dehalogenase (HAD)–like domain; RP domain; conserved Trp-637; acidic tail (AT); and intrinsically disordered regions (IDRs). Pah1, which catalyzes the Mg^2+^-dependent dephosphorylation of PA, plays a key role in the production of DAG for the synthesis of the storage lipid TAG and negatively regulates the synthesis of the PA-derived membrane phospholipids. The DAG produced by Pah1 may also be used for the synthesis of PC and PE when cells are supplemented with choline and ethanolamine, respectively. Additional roles of PA and DAG in cell physiology are indicated. More comprehensive pathways of lipid synthesis may be found in Refs. (2, 3). DAG, diacylglycerol; PA, phosphatidate; PAP, PA phosphatase; PC, phosphatidylcholine; PE, phosphatidylethanolamine; TAG, triacylglycerol.

In addition to their roles in lipid synthesis, PA and DAG participate in various cellular processes, including membrane fission and fusion (9, 10, 11, 12, 13, 14), vesicular trafficking (15, 16, 17, 18, 19), lipid signaling (5, 20, 21, 22, 23, 24, 25), and the regulation of phospholipid synthesis gene expression (26, 27, 28) (Fig. 1). The requirement of Pah1 in controlling the lipid molecules is evident in the enzyme-deficient cells, which exhibit numerous deleterious phenotypes, including aberrant nuclear membrane structure, impaired lipid droplet formation, fatty acid–induced lipotoxicity, defects in vacuole fusion and autophagy, apoptosis, and a reduced chronological lifespan (1, 7, 27, 29, 30, 31, 32, 33, 34, 35, 36, 37, 38, 39, 40, 41). The significance of PAP activity in lipid metabolism and cell physiology extends to higher eukaryotes as well. The dysfunction of Pah1 orthologs in mice and humans, known as lipins (1, 42, 43), is implicated in a range of lipid-related diseases, including lipodystrophy, rhabdomyolysis, inflammation, insulin resistance, peripheral neuropathy, osteomyelitis, congenital dyserythropoietic anemia, and type 2 diabetes (42, 44, 45, 46, 47, 48, 49, 50, 51, 52, 53, 54).

Pah1 is a peripheral membrane enzyme that exerts its PAP activity at the nuclear/ER membrane (55). Its subcellular localization for catalytic function is regulated through phosphorylation and dephosphorylation (56). The phosphorylation of Pah1 by protein kinases stabilizes the enzyme in the cytosol but prevents its interaction with the membrane (32, 57, 58, 59, 60, 61, 62, 63, 64). For its targeted membrane localization, Pah1 is recruited and dephosphorylated by the Nem1–Spo7 protein phosphatase complex in the nuclear/ER membrane (29, 32, 55, 56, 65, 66). The dephosphorylated Pah1, which freely associates with the membrane, recognizes PA, dephosphorylates it, and moves along the membrane for additional rounds of catalysis (67). Unlike the phosphorylated Pah1, its dephosphorylated form is susceptible to degradation by the 20S proteasome (68).

The function of Pah1 and its regulation depend on distinct domains and regions (69) (Fig. 1). The catalytic core of the enzyme, which is required for catalytic function, consists of the N-terminal amphipathic helix for membrane association (55), the N-LIP and haloacid dehalogenase (HAD)–like domains cofolding in the tertiary structure for PAP activity (1, 70), and the WRDPLVDID domain containing a conserved Trp-637 residue for the enzyme function *in vivo* (71, 72). The intrinsically disordered regions (IDRs) of Pah1 contain most of the phosphorylation sites for the control of the enzyme localization, activity, and stability (56), and the IDR phosphorylation is facilitated by the RP domain (73). The C-terminal acidic tail is responsible for the priming interaction with the Spo7 subunit of the Nem1–Spo7 complex for the recruitment of Pah1 to the ER membrane (65).

Pah1 belongs to the HAD-like superfamily (1, 74), a group of enzymes that catalyze the phosphoryl transfer reaction on a variety of substrates (75, 76, 77, 78). For the phosphoryl transfer chemistry, the HAD-like enzymes contain the Rossmann-like fold, a conserved catalytic structure that is featured as a central β-sheet composed of five to seven parallel β-strands surrounded by α-helices (75, 79, 80). Studies on the HAD-like enzymes other than Pah1–lipin PAPs have identified four active site motifs (74, 75, 76, 77, 78, 79, 81, 82, 83, 84, 85, 86, 87): motif I, characterized by the sequence D*X*D*X*(T/V) at the end of β-strand 1; motif II, which contains the Thr or Ser residue at the end of β-strand 2; motif III, which features a conserved Lys residue within an α-helix surrounding the central β-sheet with its side chain pointing toward the active site; and motif IV at the end of β-strand 4, which is often represented as G(D/N)*XXX*D but exhibits greater sequence variation (75).

The active site motifs I–IV of Pah1 and its orthologs were shown by sequence alignment (Fig. 2) combined with AlphaFold modeling (88, 89, 90) (Fig. 3) informed by the crystal structure of *Tetrahymena thermophila* Pah2 (91), which lacks the regulatory domains/regions of Pah1. While the motif I of Pah1 is known for its requirement for catalytic and physiological functions (70), the roles of its motifs II–IV remain unclear. In this study, we demonstrated that all four motifs of Pah1 are essential for catalytic activity and required for its cellular functions. These findings advance the understanding of the catalytic mechanism of Pah1 and provide a mechanistic basis for lipid-associated diseases caused by the mutations of human lipin PAPs in the active site residues.

**Figure 2:**
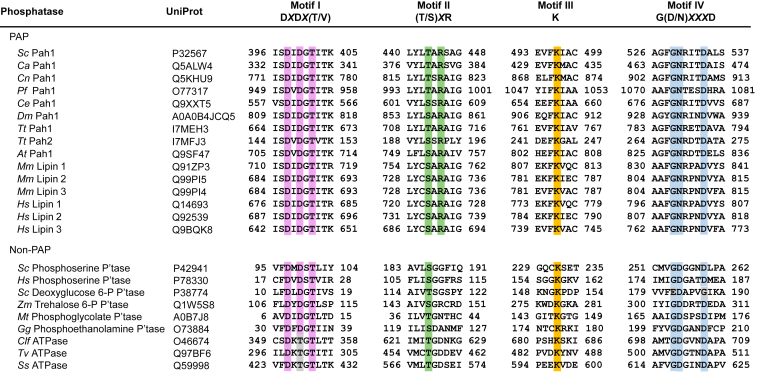
**Four conserved motifs in the HAD-like domain of PAP and non-PAP enzymes.** Clustal Omega alignment of HAD-like domain motifs in PAP and non-PAP enzymes with their UniProt accession numbers; the consensus sequence for each motif is shown above the alignment. Residue numbers are indicated at the start and end of the aligned sequences. The conserved residues of each motif are highlighted in color. *At*, *Arabidopsis thaliana*; *Ca*, *Candida albicans*; *Ce*, *Caenorhabditis elegans*; *Clf*, *Canis lupus familiaris*; *Cn*, *Cryptococcus neoformans*; *Dm*, *Drosophila melanogaster*; *Gg*, *Gallus gallus*; *Hs*, *Homo sapiens*; *Mm*, *Mus musculus*; *Mt*, *Methanothrix thermoacetophila*; *Pf*, *Plasmodium falciparum*; *Sc*, *Saccharomyces cerevisiae*; *Ss*, *Synechocystis sp. PCC 6803*; *Tt*, *Tetrahymena thermophila*; *Tv*, *Thermoplasma volcanium*; *Zm*, *Zea mays*. HAD, haloacid dehalogenase; PAP, PA phosphatase.

**Figure 3:**
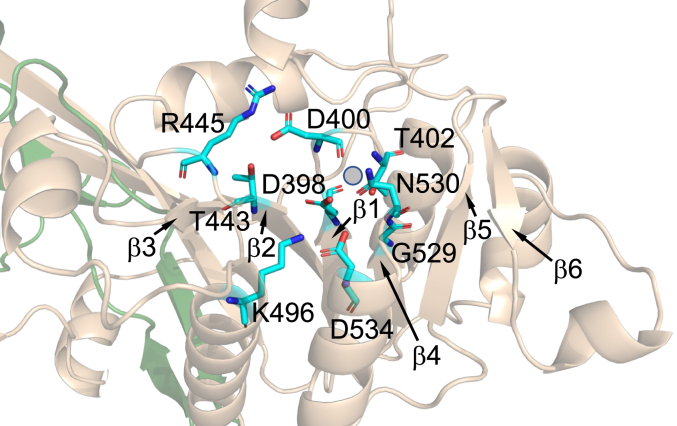
**Prediction/model of Pah1 active site architecture.** The active site of Pah1 predicted by AlphaFold (88, 89, 90) is visualized by the PyMol program. The conserved residues of active site motifs I (Asp-398 and Asp-400), II (Thr-443 and Arg-445), III (Lys-496), and IV (Gly-529, Asn-530, and Asp-534), and β-strains 1 to 6 are indicated. Mg^2+^ is depicted by the *gray circle*.

## Results

### Active site motifs I–IV of Pah1 are required for PAP activity

**Figure 4:**
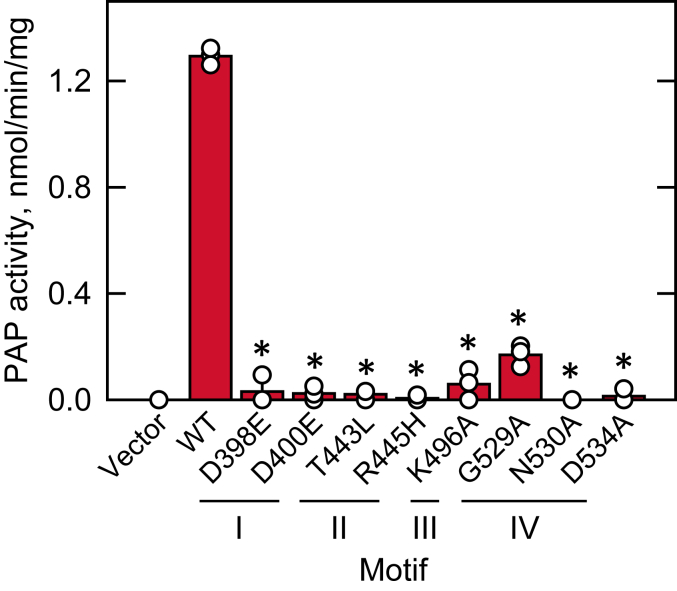
**Effect of Pah1 active site motif mutations on PAP activity.** The *pah1*Δ *app1*Δ *dpp1*Δ *lpp1*Δ (GHY66) cells were transformed with pRS415 (vector), pGH315 (*PAH1*), and pGH315 derivatives containing active site motif mutations. The yeast transformants were grown at 30 °C in SC-Leu medium to the exponential phase. Cell extracts were prepared and measured (30 μg) for PAP activity by following the release of ^32^P_i_ from [^32^P]PA. The data are means ± SD (*error bars*) from triplicate determinations; the individual data points for each experiment are shown. ∗*p* < 0.05 *versus* WT. PAP, PA phosphatase.

Sequence alignment showed that the HAD-like domain of Pah1 contains four motifs (Fig. 2), which are located within the active site that is predicted by the AlphaFold modeling (88, 89, 90) (Fig. 3). The active site motifs of Pah1 are characterized by the conserved residues, which are Asp-398, Asp-400, and Thr-402 in motif I, Thr-443 and Arg-445 in motif II, Lys-496 in motif III, and Gly-529, Asn-530, and Asp-534 in motif IV (Figures 2 and 3). Arg-445, which is not conserved in non-PAP proteins, was identified in our analysis as a conserved residue of the active site in Pah1 and its orthologs (Figs. 2 and 3). To examine the importance of the conserved residues for PAP activity, we examined their mutational effects. By site-directed mutagenesis, we generated the plasmid-borne *PAH1* alleles containing point mutations for the conserved residues (Table 1). The D398E and D400E mutations conserve the negative charge; the T443L mutation replaces the polar residue with a nonpolar hydrophobic residue; the R445H mutation conserves the positive charge; K496A mutation replaces the positive charge with a nonpolar residue; G529A mutation introduces steric hindrance; the N530A mutation replaces the polar uncharged residue to a nonpolar residue; and D534A mutation replaces the negative charge to the nonpolar residue. In particular, the T443L and R445H mutations were made to mirror human lipin–related disease mutations. The mutant alleles were expressed in the *pah1*Δ *app1*Δ *dpp1*Δ *lpp1*Δ strain (8), which lacks all the PAP-encoding genes, *PAH1* (1), *APP1* (8), *DPP1* (92), and *LPP1* (93). The use of the quadruple mutant ensured that cellular PAP activity was solely attributed to the plasmid-borne *PAH1* allele. The exponential phase cell extracts were prepared from the PAP-deficient strain expressing Pah1 active site mutants and analyzed for PAP activity by measuring the release of ^32^P_i_ from ^32^P-labeled PA (94). In the PAP assay, the substrate PA was delivered to the assay mixture as a uniform Triton X-100/PA-mixed micelle to mimic the membrane surface for catalysis (95). As expected, the PAP-deficient strain exhibited PAP activity (1.2 nmol/min/mg) by the expression of WT Pah1 (Fig. 4). Compared with the WT enzyme, active site mutants in the motifs I, II, and III showed almost complete lack of PAP activity. Of the mutants in the motif IV, N530A and D534A were inactive, whereas G529A showed a very weak activity (0.17 nmol/min/mg), which is sevenfold lower than that of the WT enzyme (Fig. 4). These results indicate that the conserved residues in the active site motifs I–IV are crucial for PAP activity.

**Table 1:** Plasmids and strains used in this study

Plasmid or strain	Genotype or relevant characteristics	Source or reference
Plasmid		
pRS415	Single-copy *E. coli*/*S. cerevisiae* shuttle vector with *LEU2*	(139)
*Derivative*		
pGH315	*PAH1* with native promoter in pRS415	(59)
pGH315-D398E	pGH315 with the D398E mutation in *PAH1*	(98)
pGH315-D400E	pGH315 with the D400E mutation in *PAH1*	(98)
pGH315-T443L	pGH315 with the T443L mutation in *PAH1*	This study
pGH315-R445H	pGH315 with the R445H mutation in *PAH1*	This study
pGH315-K496A	pGH315 with the K496A mutation in *PAH1*	This study
pGH315-G529A	pGH315 with the G529A mutation in *PAH1*	This study
pGH315-N530A	pGH315with the N530A mutation in *PAH1*	This study
pGH315-D534A	pGH315 with the D534A mutation in *PAH1*	This study
YCplac33	Single-copy *E. coli*/*S. cerevisiae* shuttle vector with *URA3*	(140)
*Derivative*		
YCplac33-SEC63-GFP	YCplac33 with *SEC63-GFP* fusion	(33)
pET-15b	*E. coli* IPTG-inducible expression vector with N-terminal His_6_-tag fusion	Novagen
*Derivative*		
pGH313	pET-15b with *PAH1* coding sequence	(1)
pGH313-D398E	pGH313 with the D398E mutation in *PAH1*	(70)
pGH313-T443L	pGH313 with the T443L mutation in *PAH1*	This study
pGH313-K496A	pGH313 with the K496A mutation in *PAH1*	This study
pGH313-N530A	pGH313 with the N530A mutation in *PAH1*	This study
pGH313-ΔAH	pGH313 with the ΔAH (Δ2-18) mutation in *PAH1*	This study
Strain		
*S. cerevisiae*		
RS453	*MAT***a***ade2-1 his3-11,15 leu2-3112 trp1-1 ura3-52*	(141)
*Derivative*		
SS1026	*pah1*Δ*::TRP1*	(29)
W303-1A	*MAT***a***ade2-1 can1-100 his3-11,15 leu2-3112 trp1-1 ura3-1*	(142)
*Derivative*		
GHY66	*pah1*Δ*::URA3 app1*Δ*::natMX4 dpp1*Δ*::TRP1/Kan*^*r*^*lpp1*Δ*::HIS3/Kan*^*r*^	(8)
*E. coli*		
DH5⍺	F^-^ Φ80 *lacZ*ΔM15Δ (*lacZYA-argF*)U169 *deoR rec*A1 *end*A1 *hsd*R17 (r_k_^-^ m_k_^+^) *pho*A *sup*E44 λ^−^*thi*-1 *gyr*A96 *rel*A1	(124)
NiCo21 (DE3)pLysSRARE2	*can::CBD fhuA2 [Ion] ompT gal (λ DE3) [dcm] amA::CBD sly::CBD glmS6Ala* Δ*hsdS* λ *DE3* = λ *sBamHIo* Δ*EcoRI-B int::(lacI::PlacUV5::T7 gene1) i21* Δ*nin5* pLysSRARE2	New England Biolabs

### Pah1 active site mutants exhibit reduced stability and phosphorylation

**Figure 5:**
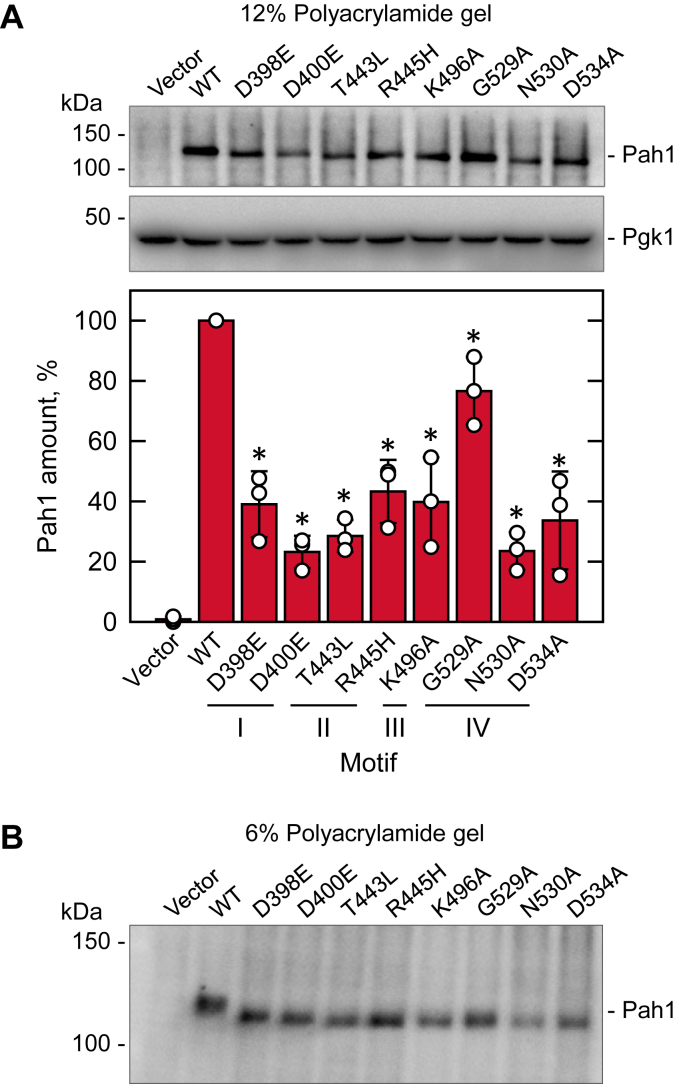
**Cellular level and phosphorylation state of Pah1 active site motif mutants.** The *pah1*Δ *app1*Δ *dpp1*Δ *lpp1*Δ (GHY66) cells were transformed with pRS415 (vector), pGH315 (*PAH1*), and pGH315 derivatives containing the active site motif mutations. The yeast transformants were grown at 30 °C in SC-Leu medium to the exponential phase. Samples (30 μg) of the cell extracts were separated by SDS-PAGE using the 12% (*A*) and 6% (*B*) polyacrylamide gels, transferred to a PVDF membrane, and probed with anti-Pah1 (*A* and *B*) and anti-Pgk1 (*A*) antibodies. The relative amounts of Pah1 (*A*) were determined by image analysis using iBright 1500 Imager and iBright Analysis software. The positions of Pah1, Pgk1, and molecular mass standards are indicated. The immunoblots shown are representative of three independent experiments. The data are means ± SD (*error bars*) from triplicate determinations; the individual data points for each experiment are shown. ∗*p* < 0.05 *versus* WT.

To confirm that activity deficiency of the Pah1 mutants was not because of the lack of the enzymes, we examined their expression by immunoblot analysis with anti-Pah1 antibody. As described previously (96), WT Pah1 was detected from the extracts of the exponential-phase cells used for PAP assay (Fig. 5). Likewise, its active site mutants were all detected but at a lower level (Fig. 5*A*). Since WT and mutant forms of Pah1 are expressed from the same gene promoter, the different enzyme levels are most likely because of a difference in their stability. In addition to their reduced levels, the active site mutants showed a faster electrophoretic mobility, which was readily noticeable in a low-percentage (6%) polyacrylamide gel (Fig. 5*B*). The electrophoretic mobility of Pah1 reflects its phosphorylation state (66, 73), and the faster mobility of the active mutants suggests that they are less phosphorylated than the WT enzyme. The phosphorylation state of Pah1 is intimately related to its stability, and its phosphorylation-deficient form is unstable in the cell (96). Accordingly, these results indicate the active site mutants of Pah1, which lack PAP activity, are less phosphorylated, and unstable.

### Pah1 active site mutants fail to complement the *pah1*Δ phenotypes

Loss of Pah1 function in *S. cerevisiae* results in a variety of cellular changes (reviewed in Ref. (5)). Prominent phenotypes of the *pah1*Δ mutant include a decrease in the TAG level and a reciprocal increase in the level of membrane phospholipids (1, 7, 37). The reduced TAG synthesis caused by a defect in DAG production correlates with a significantly reduced number of cytoplasmic lipid droplets (97, 98). In contrast, the increased level of membrane phospholipids, which is caused by the accumulation of PA that serves as a phospholipid precursor and as a transcriptional regulator for the derepression of phospholipid biosynthetic genes (2, 5, 26), is associated with the aberrant expansion of the nuclear/ER membrane (29, 70). Moreover, yeast cells lacking Pah1 function manifest diverse physiological defects that include a shortened chronological lifespan, apoptotic cell death in stationary phase, and an inability to grow at high temperature and on nonfermentable carbon sources (1, 29, 30, 36, 98). To examine whether the active site mutants of Pah1 complement the *pah1*Δ phenotypes, they were expressed on a single-copy plasmid in the *pah1*Δ or *pah1*Δ *app1*Δ *dpp1*Δ *lpp1*Δ cells (Table 1).

**Figure 6:**
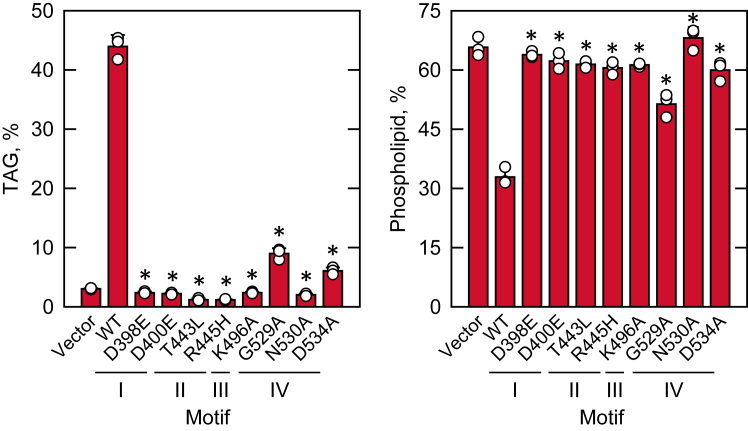
**Effect of Pah1 active site motif mutations on the synthesis of TAG and phospholipids.** The *pah1*Δ (SS1026) cells were transformed with pRS415 (vector), pGH315 (*PAH1*), and pGH315 derivatives containing the active site motif mutations. The transformants were grown at 30 °C to the stationary phase in SC-Leu medium containing 1 μCi/ml [2-^14^C]acetate. Radiolabeled lipids were extracted, separated by one-dimensional TLC, and quantified by phosphorimaging coupled with an image analysis by ImageQuant. The levels of TAG and phospholipids in each strain were normalized to its total ^14^C-labeled chloroform-soluble fraction. The data are means ± SD (*error bars*) from biological triplicates. The individual data points are also shown. ∗*p* < 0.05 *versus* WT. TAG, triacylglycerol.

To assess the effect of the Pah1 mutants on lipid synthesis, the *pah1*Δ transformants were labeled to steady state with [2-^14^C]acetate and harvested in the stationary phase, then their lipids were extracted and analyzed by TLC (Fig. 6). Stationary-phase cells were examined for lipid analysis as the effect of Pah1 is pronounced when the TAG level is highest (1, 7). Consistent with previous findings (1, 7), *pah1*Δ cells harboring an empty vector showed a very low level of TAG (3%) but a significantly high level of phospholipids (66%). The expression of WT Pah1 in *pah1*Δ cells resulted in a substantial increase in the TAG level (44%) with a reciprocal decrease in the level of phospholipids (33%). In contrast to the WT enzyme, its active site mutants, except for G529A (9%) and D534A (6%), did not significantly increase the TAG level of *pah1*Δ cells.

**Figure 7:**
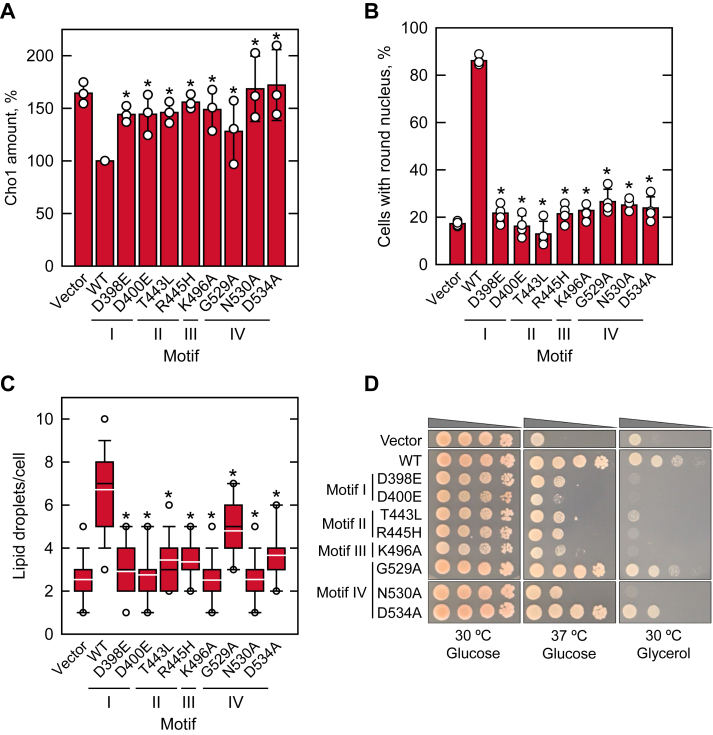
**Effect of Pah1 active site motif mutations on the complementation of the *pah1*Δ physiological defects.***A*, *pah1*Δ *app1*Δ *dpp1*Δ *lpp1*Δ (GHY66) cells transformed with pRS415 (vector), pGH315 (*PAH1*), or pGH315 derivatives were grown in SC-Leu medium to the exponential phase. The expression of Cho1 was detected from cell extracts (10 μg) by immunoblot analysis with anti-Cho1 antibody, quantified by ImageQuant analysis, and normalized to the level of WT Pah1. *B*, *pah1*Δ cells harboring YCplac33-*SEC63-GFP* were transformed with pRS415 (vector), pGH315 (*PAH1*), or a pGH315 derivative. The yeast transformants were grown in SC-Leu-Ura medium to the exponential phase, examined by fluorescence microscopy for the signal of the ER marker Sec63-GFP, and scored for normal nuclear morphology from ≥200 cells. *C*, *pah1*Δ cells transformed with pRS415 (vector), pGH315 (*PAH1*), or a pGH315 derivative were grown to the stationary phase and stained with BODIPY 493/503. Lipid droplets were visualized by fluorescence microscopy from ≥300 cells. The *black* and *white lines* in the *box plot* are the median and mean values, respectively, and the *white circles* are the outlier data points of the 5th and 95th percentile. *D*, *pah1*Δ cells transformed with pRS415 (vector), pGH315 (*PAH1*), or a pGH315 derivative were grown to saturation and adjusted to an absorbance of 0.67 at 600 nm, followed by 10-fold serial dilution. About 5 μl of the diluted cultures were spotted on SC-Leu plates with 2% glucose (*left* and *center*) or 2% glycerol (*right*) and incubated at 30 °C (*left* and *right*) or 37 °C (*center*). The cell growth on glucose and glycerol plates was scored after incubation for 3 and 4 days, respectively. The data in *A*, *B*, and *C* are means ± SD (*error bars*) from triplicate determinations. *∗p* < 0.05 *versus* the WT. ER, endoplasmic reticulum.

The *pah1*Δ mutant, which contains the elevated level of PA (1, 28, 37, 99), exhibits the derepression of phospholipid biosynthetic genes (*e.g.*, *INO1*, *CHO1*, *OPI3*) containing the UAS_INO_ *cis*-acting element in the promoter (27, 28, 29) through the Opi1/Ino2–Ino4 (Henry) regulatory circuit (2, 3, 5, 26, 100). In this regulation, the Opi1 repressor is sequestered at the nuclear/ER membrane through its binding to PA and does not inhibit the activator function of the Ino2–Ino4 complex in the transcription of the UAS_INO_-containing genes (101). To determine the Pah1-mediated regulation of phospholipid biosynthetic gene expression, we examined the level of the *CHO1*-encoded PS synthase. Consistent with the previous finding (27), the elevated level of Cho1 in *pah1*Δ cells was reduced (∼1.6-fold) by the expression of WT Pah1 (Fig. 7*A*). However, little change was observed in the Cho1 level by the active site mutants (Fig. 7*A*).

The increased synthesis of membrane phospholipids in the *pah1*Δ mutant is responsible for the nuclear/ER membrane expansion leading to an aberrant nuclear morphology (27, 29, 70). The nuclear morphology of *pah1*Δ cells harboring WT Pah1 or its active site mutant was monitored by the coexpression of the nuclear/ER marker Sec63-GFP. While the expression of WT Pah1 in *pah1*Δ cells restored normal, round nuclear morphology, the complementation effect was not clearly shown by the active site mutants (Fig. 7*B*). As previously reported (72, 102, 103), the expression of WT Pah1 in *pah1*Δ cells restored the formation of cytoplasmic lipid droplets (∼7 lipid droplets/cell). However, the expression of the active site mutants in motifs I–IV, which lack PAP activity, did not significantly increase the lipid droplets (2–3 lipid droplets/cell). The G529A mutant exhibiting weak PAP activity showed a partial effect on lipid droplet formation (∼5 lipid droplets/cell) (Fig. 7*C*). Moreover, the active site mutants, except for G529A and D534A, did not restore the growth defect of *pah1*Δ cells at 37 °C or on growth medium containing glycerol, a nonfermentable carbon source (Fig. 7*D*).

### Effect of Pah1 active site mutations on the kinetics of PAP activity

**Figure 8:**
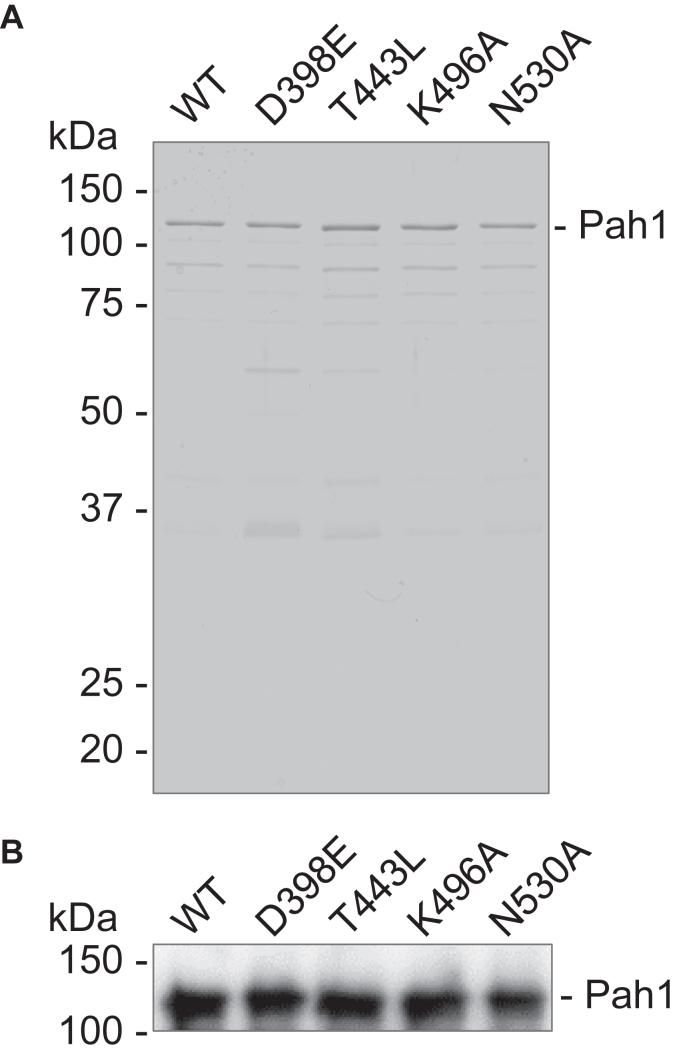
**Purification of Pah1 and its active site motif mutants.** The His_6_-tagged Pah1 and its catalytic motif mutants were expressed in *Escherichia coli* and purified by immobilized metal affinity chromatography. *A*, samples of the purified enzymes were subjected to SDS-PAGE (12% polyacrylamide gel) and stained with Coomassie blue. *B*, the purified enzyme preparations were subjected to immunoblot analysis with anti-Pah1 antibody. The positions of Pah1 and molecular mass standards are indicated. The data shown are representative of two experimental replicates.

We further investigated Pah1 active site mutants, which do not exhibit the cellular function, for PAP activity in a more defined system. The His_6_-tagged Pah1 and its D398E (motif I), T443L (motif II), K496A (motif III), and N530A (motif IV) mutants were heterologously expressed in *Escherichia coli* and purified by immobilized metal affinity chromatography and ion exchange chromatography. The *E. coli*-expressed Pah1 lacks phosphorylation (59), mimicking the dephosphorylated form of the enzyme, which is functional on the nuclear/ER membrane of *S. cerevisiae* (56, 104). Moreover, the unphosphorylated form of Pah1 can associate with the membrane in the absence of Nem1–Spo7, the protein phosphatase that catalyzes the dephosphorylation of Pah1 (67). The purified WT and the active site mutant forms of Pah1 were confirmed by SDS-PAGE (Fig. 8*A*) and immunoblot analysis with anti-Pah1 antibody (Fig. 8*B*).

**Figure 9:**
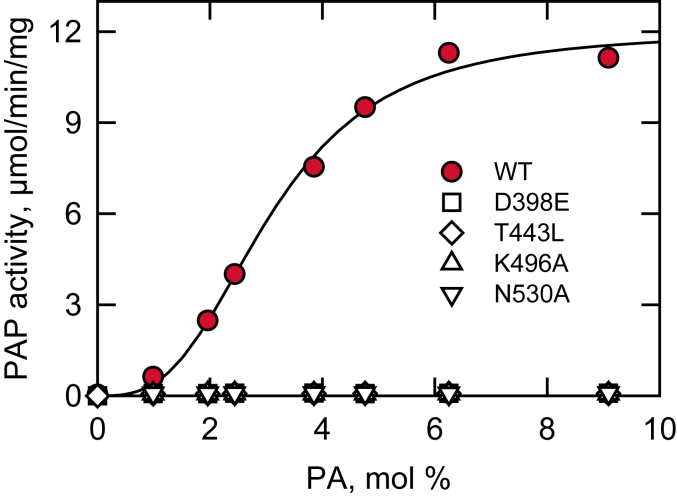
**Effect of Pah1 active site motif mutations on the kinetics of PAP activity.** The PAP activity of WT Pah1 and its catalytic motif mutants was measured as a function of the PA surface concentration (mol %); the enzyme activity was measured by following the release of ^32^P_i_ from [^32^P]PA. The surface concentration of PA (mol %) was adjusted by maintaining the molar concentration of PA at 0.2 mM and varying the molar concentration of Triton X-100 (106). The data are means ± SD (*error bars*) from triplicate assays. Some error bars are contained within the data symbols. PAP, PA phosphatase.

The purified WT and active site mutant enzymes were examined for their kinetic parameters of PAP activity. In the PAP assay, Triton X-100 is used to create mixed micelles with PA, providing a surface for catalysis (95, 105, 106). Therefore, PA concentration is expressed as a surface concentration (mol %) rather than a molar concentration (95). This system ensures that the measured PAP activity is dependent on the surface concentration of PA and independent of its molar concentration (95). Consistent with previous findings (1, 70), WT Pah1 displayed positive cooperative kinetics with respect to the PA surface concentration, with a *V*_max_ of 12 μmol/min/mg, a *K*_*m*_ for PA of 3 mol%, and a Hill number of 3 (Fig. 9). However, the mutant enzymes did not show PAP activity at all the concentrations examined, and their kinetic parameters could not be determined (Fig. 9).

### Active site mutations of Pah1 do not affect its overall structure and interaction with liposomes

**Figure 10:**
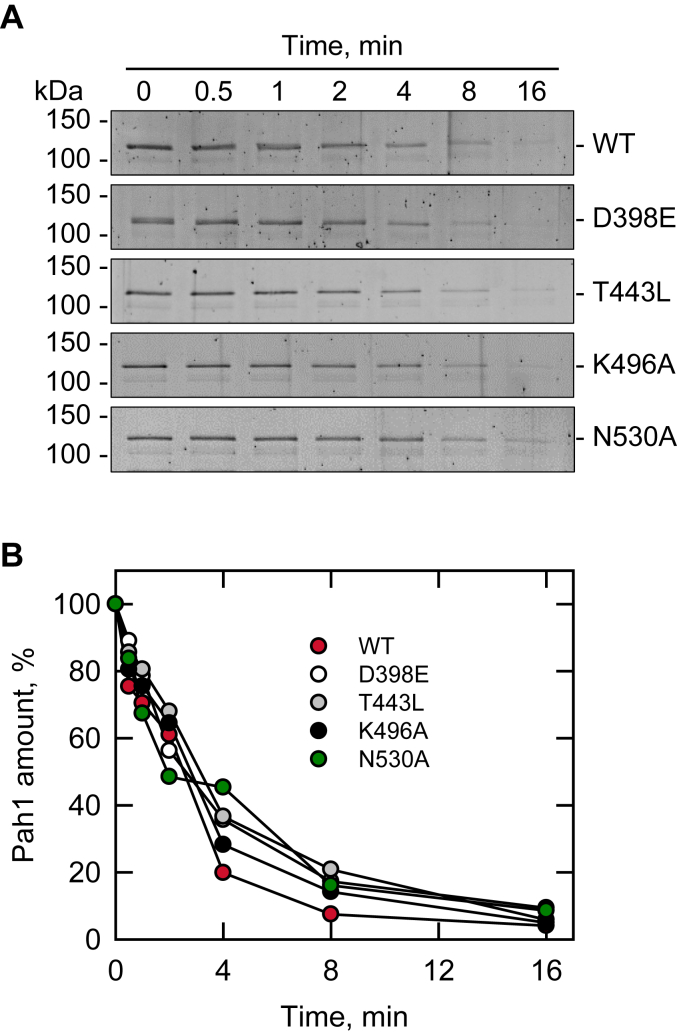
**Limited chymotrypsin digestion of Pah1 and its active site motif mutants.***A*, samples (0.1 μg) of purified Pah1 and its active site motif mutants were incubated at 23 °C for the indicated periods with 25 ng of chymotrypsin in phosphate-buffered saline (pH 7.4). The digestion mixtures were separated by SDS-PAGE (12% polyacrylamide gel) and stained with SYPRO Ruby. The full-length position of Pah1 and its mutant forms and molecular mass standards are indicated. The data are representative of duplicate experiments. *B*, the amounts of full-length Pah1 and its mutant forms remaining after chymotrypsin treatment were determined by image analysis using iBright 1500 Imager and iBright Analysis software. The data are the average of duplicate experiments.

AlphaFold modeling (88, 89, 90) shows that the overall structure of Pah1 is not significantly affected by the active site mutations D398E, T443L, K496A, and N530A. To determine whether these mutations cause an overall structural change, we performed a limited proteolysis assay (107, 108). The WT and mutant forms of the *E. coli*-expressed, unphosphorylated Pah1 were incubated with chymotrypsin for different periods, and the protein digests were separated by SDS-PAGE (Fig. 10*A*). The analysis of the full-length Pah1 showed that WT and its active mutants were digested in a very similar extent (Fig. 10*B*). This result suggests that the overall structure of dephosphorylated Pah1, which functions on the nuclear/ER membrane, is not affected by the active site mutations D398E, T443L, K496A, and N530A.

**Figure 11:**
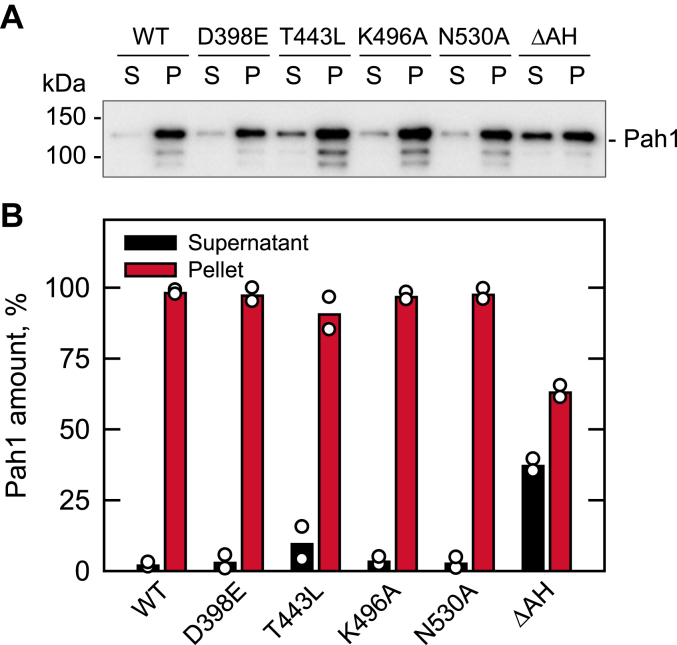
**Liposome interaction of Pah1 and its active site motif mutants.** WT and mutant forms of Pah1 (0.1 μg) were incubated for 15 min at 30 °C with (10 mM phospholipid) PC/PE/PI/PS/PA liposomes. Following the incubation, liposomes were precipitated by centrifugation at 100,000*g* for 1 h at 4 °C. The pellet (*P*) fraction was suspended in buffer to the same volume as that of the supernatant (*S*) fraction, and equal volumes of the fractions were subjected to SDS-PAGE, followed by immunoblot analysis with anti-Pah1 antibody. *A*, the immunoblot shown is representative of two experiments. The positions of Pah1 and molecular mass standards are indicated. *B*, the amounts of Pah1 associated with the liposome were quantified by image analysis using iBright 1500 Imager and iBright Analysis software. The bars are means of the experimental replicates; the individual data points are shown. PA, phosphatidate; PC, phosphatidylcholine; PE, phosphatidylethanolamine; PI, phosphatidylinositol; PS, phosphatidylserine.

We next examined the interaction of unphosphorylated Pah1 with liposomes whose phospholipid contents PC/PE/PI/PS/PA (33.75:22.5:22.5:11.25:10 mol%) reflect the composition of the nuclear/ER membrane and the subcellular location where dephosphorylated Pah1 associates for catalytic activity (67, 109). To assess only its membrane association, Pah1 was incubated with liposomes in the absence of the cofactor Mg^2+^ required for catalysis (1, 105). Liposomes were collected by centrifugation and examined for the presence of Pah1 by immunoblot analysis with anti-Pah1 antibody. As described previously (67), WT Pah1 was found exclusively in the pellet of liposomes (Fig. 11). Similarly, the active site mutants were also found almost exclusively in the pellet fraction. As expected, Pah1 mutant (ΔAH), which is defective in membrane association because of the lack of the amphipathic helix (55), showed a great reduction in the liposomal pellet (Fig. 11). These results indicate that the active site mutations of Pah1 do not affect its ability to interact with the membrane.

## Discussion

Pah1, which controls the levels of PA and DAG at the nuclear/ER membrane, plays a major role not only in lipid synthesis but also in diverse cellular processes (29, 32, 55, 56, 65, 66, 102, 110, 111). Pah1 belongs to the Mg^2+^-dependent HAD-like phosphatase superfamily, characterized by the D*X*D*X*(T/V) motif (1, 70, 74). This superfamily encompasses a broad range of phosphatases with diverse substrate specificities (74). Research on other HAD-like family members suggests a four-motif active site, with the D*X*D*X*(T/V) sequence designated as motif I (74). Studies on phosphoserine phosphatase, a non-Pah–lipin PAP enzyme, have demonstrated the importance of all four motifs for its activity (82). In motif I, the first Asp acts as a nucleophile, forming a phosphoryl–aspartate intermediate, whereas the second Asp deprotonates a water molecule to facilitate hydrolysis of this intermediate (76, 85). Motifs II and III are thought to stabilize the reaction intermediate (75), and motif IV coordinates the essential Mg^2+^ ion (75, 82). Sequence alignments show that Pah1, like its mammalian lipin homologs, contains all four motifs within its HAD-like domain (Fig. 2). In this work, we identified the active site motifs I–IV of Pah1 and its orthologs (Fig. 2) and examined Pah1 as a model for the Mg^2+^-dependent PAPs to show that the conserved residues of the motifs are crucial for PAP activity and the cellular functions of the enzyme.

Our findings demonstrated that the Pah1 mutations in the active site motifs I–IV abolish PAP activity without affecting the enzyme interaction with the liposome membrane. The catalytically inactive Pah1 failed to rescue the *pah1*Δ phenotypes, including reduced levels of TAG and lipid droplets, aberrant nuclear morphology, increased expression of Cho1 PS synthase, and growth defects at high temperature and on glycerol medium. Thus, the conserved residues of Pah1 in the motifs I–IV are essential for PAP activity and thereby for the control of lipid synthesis, gene regulation, and other cellular processes. The proteolysis analysis suggests that the overall structure of Pah1 is not significantly affected by its active site mutations. Interestingly, however, the Pah1 mutants were less abundant in the cell and exhibited a reduced phosphorylation state. The lower cellular level of the mutant protein is likely to result from an increased susceptibility to proteolysis under the reduced state of phosphorylation (68, 96). It is yet unclear why the active site mutants are in a lower phosphorylation state. Pah1 mutations that do not directly affect PAP activity also affect the phosphorylation state and stability of the enzyme (71, 72). Thus, we cannot conclude that PAP activity *per se* regulates Pah1 phosphorylation. Considering that the cells lacking Pah1 PAP activity exhibit diverse physiological changes, the reduced phosphorylation of mutant Pah1 may result from a decreased protein kinase activity or by an increased protein phosphatase activity in the PAP-deficient cell, which is not complemented by the expression of the nonfunctional enzyme.

We identified Arg-445 as a motif II residue conserved in Pah1 and its orthologs but not in other HAD-like enzymes (Fig. 2). The conservation of the arginine residue in Pah1–lipin PAPs could stem from a difference in enzyme structure requirements and/or its substrate specificity. Pah1–lipin PAPs contains the N-LIP domain (42), which is not present in other members of the HAD-like enzymes such as phosphoserine phosphatase (82). The N-LIP domain and the HAD-like domain cofold to form a two-domain structure as the catalytic core of PAP (42, 69, 70, 91). Unlike the HAD-like domain, which contains the active site motifs, the role of the N-LIP domain in PAP activity is unclear. In kinetic analysis, a Pah1 variant containing the G80R mutation in the N-LIP domain exhibits a greater reduction in *V*_max_ when compared with a change in *K*_m_ (70), suggesting the contribution of the N-LIP domain to catalysis more than to substrate binding. While containing the N-LIP domain, Pah1–lipin PAPs lack a cap domain (69, 91), a mobile element influencing substrate specificity in many HAD-like phosphatases (75, 112). Protein modeling (113) indicates that the active site of Pah1 is smaller than that of non-PAP HAD-like enzymes (114), suggesting that it can accommodate only the phosphate group of PA. Indeed, the acyl chain composition of PA has no effect on the catalytic activity of Pah1 and lipin 1 (67, 115). Unlike the non-PAP enzymes acting on water-soluble substrates (74, 75, 76, 77, 78), Pah1–lipin PAPs utilize water-insoluble PA as a substrate (1, 67, 115). Further research is needed to determine whether structural differences are associated with the role of the conserved arginine residue of Pah1–lipin PAP enzymes.

Given that Lys-496 in motif III is crucial for the catalytic activity of Pah1, any modification of the active site is likely to abolish the cellular function of the enzyme. As reported here, other studies have shown that Pah1 mutated at Lys-496 is nonfunctional and does not complement the *pah1*Δ phenotypes, such as irregular nuclear morphology and reduced amounts of TAG and lipid droplets (116, 117). While the functional deficiency of the K496A mutant is caused by the lack of PAP activity, Laframboise *et al.* (117) attributed the mutational effect to the defect of the enzyme in localizing to the inner nuclear membrane in association with the lack of the lysine acetylation. The acetylation of Lys-496 was indicated by the analysis of Pah1 expressed heterologously in human embryonic kidney 293T cells overexpressing the Esa1 acetyltransferase (116). Despite this finding, the acetylation of Pah1 at Lys-496 is yet unclear in a native physiological condition as proteomic analysis of *S. cerevisiae* does not show this lysine to be acetylated (118). In addition, Li *et al.* (116) erroneously described that Lys-496 in the HAD-like domain of Pah1 corresponds to Lys-425 in the IDR of lipin 1. Lys-425 is one of the two residues (Lys-425 and Lys-595) that are acetylated by TIP60 acetyltransferase in response to fatty acid stimulation (116). The acetylation of Lys-425 and Lys-595 in lipin 1 does not affect its PAP activity but facilitates the translocation of the enzyme to the ER membrane for catalytic function, contributing to TAG synthesis and lipid droplet formation (116). In the optimal sequence alignment (42), the human lipin 1 residue corresponding to the Pah1 Lys-496 in the HAD-like domain is Lys-776 (Fig. 2), which is not subject to acetylation (116). Pah1 contributes to TAG synthesis and lipid droplet formation through its catalytic function mainly at the nuclear/ER membrane, not at the inner nuclear membrane (29, 32, 55, 56, 65, 66, 67). Accordingly, the role of Lys-496 as an acetylation site for the localization control of Pah1 is intriguing but remains uncertain.

The motif IV residues (Gly-529, Asn-530, and Asp-534) were also shown to be important for PAP activity and the physiological functions of the enzyme. Of the three residues, Asn-530 showed the strongest mutational effect. Like Asp-179 (motif IV) of phosphoserine phosphatase (82), Asn-530 is predicted to play a role in the coordination of Mg^2+^ in the active site (Fig. 3). Interestingly, the G529A mutant, which retained limited PAP activity in the cell extract, and the D534A mutant, which showed essentially no enzymatic activity in the extract, mostly complemented growth of *pah1*Δ cells at 37 °C and on glycerol-containing medium. The expression of both mutants slightly increased the TAG level of *pah1*Δ cells when compared with the vector control. Since TAG production requires Pah1 PAP activity, this indicated that the G529A and D534A mutant enzymes retain a very low level of PAP activity that is sufficient to complement these particular *pah1*Δ mutant phenotypes.

The dependence of Pah1 function on the active site motifs provides an insight into the mechanistic basis for the lipin-related diseases in humans. Specifically, three conserved residues are of interest: Thr-443 and Arg-445 in motif II of Pah1 correspond to Ser-734 of lipin 2 (UniProt no.: Q92539) and Arg-725 of lipin 1 (UniProt no.: Q14693), respectively, whereas Gly-529 in motif IV of Pah1 corresponds to Gly-799 of lipin 1 (UniProt no.: Q14693) (Fig. 2). The S734L mutation in lipin 2 (motif II), which eliminates PAP activity (49), causes Majeed syndrome, an inflammatory disorder characterized by recurrent osteomyelitis, fever, dyserythropoietic anemia, and skin inflammation (53, 119). Mutations R725H (motif II) and G799R (motif IV) in lipin 1 cause rhabdomyolysis (120), marked by myoglobinuria (121). Notably, the motif II mutations in lipins 1 and 2 result in distinct lipin-related diseases, which may be based on their differential tissue expression (43) and thus different levels of PAP activity. In *T. thermophila* Pah2 (UniProt no.: I7MFJ3), mutations S191L (motif II) and G267R (motif IV) abolish PAP activity (91), further highlighting the importance of the conserved active site residues whose mutation in human lipins leads to a disease condition.

## Experimental procedures

### Reagents

All chemicals were reagent grade. Growth medium was sourced from Difco Laboratories. Enzymes and reagents for DNA manipulations came from New England Biolabs. Carrier DNA for yeast transformations was purchased from Clontech. Qiagen was the source of nickel–nitrilotriacetic acid agarose resin and kits for plasmid and DNA gel extractions. Roche was the source for EDTA-free cOmplete ULTRA protease inhibitor tablets. Ampicillin, nucleotides, silica gel TLC plates, ATP, Triton X-100, and bovine serum albumin were acquired from Millipore–Sigma. Bio-Rad supplied the DNA size ladders, molecular mass protein standards, and reagents used for electrophoresis and immunoblotting. InstantBlue Coomassie protein stain was purchased from Expedeon. Lipids were purchased from Avanti Polar Lipids. Radiochemicals and scintillation counting supplies were acquired from Revvity and National Diagnostics, respectively. Q-Sepharose, Protein A-Sepharose CL-4B, polyvinylidene difluoride membrane, and enhanced chemifluorescence substrate were acquired from GE Healthcare. Thermo Fisher Scientific supplied Malachite green, BODIPY 493/503, and SYPRO Ruby.

### Antibodies

Rabbit polyclonal antibodies were generated against the peptide TSIDKEFKKLSVSKAGA (residues 778–794) of Pah1 (59) and MVESDEDFAPQEFPH (residues 1–15) of Cho1 (122). The IgG fraction of the antibodies was purified by affinity chromatography (123) with Protein A-Sepharose CL-4B and used for immunoblot analyses. Mouse anti-Pgk1 antibody was from Abcam (product number: ab113687; lot number: 2101050637). Goat anti-rabbit IgG antibody conjugated with alkaline phosphatase was from Thermo Fisher Scientific (product number: 31340; lot number: NJ178812). Goat anti-mouse IgG antibody conjugated with alkaline phosphatase was from Millipore–Sigma (product number: A3562; lot number: SLBG1482V).

### Plasmids, strains, and growth conditions

The plasmids and strains used in this study are listed in Table 1. The isolation of plasmid DNA, PCR amplification, restriction enzyme digestion, and DNA ligation were performed by standard methods (124, 125, 126). Plasmids pGH315 and pGH313 were used for the expression of Pah1 and its mutants in *S. cerevisiae* and *E. coli*, respectively. The derivatives of pGH315 and pGH313 containing the *PAH1* mutations in the active site motifs I–IV and amphipathic helix were constructed by the PCR-mediated site-directed mutagenesis (102), and the mutations were confirmed by DNA sequencing. *E. coli* strain DH5α was used for plasmid propagation (124), and the strain NiCo21(DE3)pLysS RARE2 (New England Biolabs) was used for heterologous expression of Pah1. *E. coli* was grown at 37 °C in lysogeny broth media (1% tryptone, 0.5% yeast extract, 1% NaCl, pH 7.0), and its plasmid transformant was selected with appropriate antibiotics. The *S. cerevisiae* strains *pah1*Δ (SS1026) and *pah1*Δ *app1*Δ *dpp1*Δ *lpp1*Δ (GHY66) were used for the expression of plasmid-borne *PAH1* and its mutant alleles. *S. cerevisiae* transformants were grown at 30 °C in synthetic complete media lacking an appropriate nutrient for plasmid maintenance. Plasmid transformations of *E. coli* (124) and *S. cerevisiae* (127) were performed by standard methods. The growth of *E. coli* and *S. cerevisiae* in liquid media was monitored by measuring the absorbance at 600 nm. Solid growth media contained 1.5% and 2% agar for *E. coli* and *S. cerevisiae*, respectively.

### Preparation of yeast cell extracts

All steps were conducted at 4 °C. The exponential-phase cultures (absorbance at 600 nm = 0.5) of *S. cerevisiae pah1*Δ *app1*Δ *dpp1*Δ *lpp1*Δ expressing *PAH1* and its mutant alleles were lysed by mechanical disruption with glass beads (0.5-mm diameter) in the buffer containing 50 mM Tris–HCl (pH 7.5), 10 mM 2-mercaptoethanol, 100 mM sucrose, and the EDTA-free protease inhibitor cocktail (94). The lysate was centrifuged at 1500*g* for 10 min to remove the unbroken cells and glass beads, and the supernatant was used as cell extract.

### Purification of Pah1

*E. coli* NiCo21(DE3)pLysS RARE2 harboring the pGH313 or its derivatives was grown in lysogeny broth containing chloramphenicol (34 μg/ml) and ampicillin (100 μg/ml) until absorbance at 600 nm reaches 1.0 and was added with 1 mM isopropyl-β-D-thiogalactoside to express the His_6_-tagged Pah1 (1) and its mutants. The epitope-tagged proteins were isolated from the induced culture by immobilized metal affinity chromatography with nickel–nitrilotriacetic acid agarose. The affinity-purified enzymes were further purified by anion exchange chromatography with Q-Sepharose (1, 66) and stored at −80 °C until use.

### Preparation of Triton X-100/PA-mixed micelles

PA dissolved in chloroform was dried *in vacuo*, followed by its suspension in Triton X-100 to prepare Triton X-100/PA-mixed micelles (94). The molar percent of PA in the Triton X-100/PA-mixed micelle was calculated by the formula: mol %_PA_ = 100 × [PA (molar)]/([PA (molar)] + [Triton X-100 (molar)]). The PA concentration was kept below 15 mol% to ensure that the structure of the Triton X-100/PA-mixed micelles was similar to that of pure Triton X-100 micelles (128, 129).

### Preparation of liposomes

Unilamellar liposomes, which are composed of PC/PE/PI/PS/PA (33.75:22.5:22.5:11.25:10 mol%), were prepared by the extrusion method of MacDonald *et al.* (130). Briefly, the phospholipid mixture (10 mM) dissolved in chloroform was dried under a stream of nitrogen to form a thin film, and the residual solvent was removed *in vacuo* for 30 min. Dried phospholipids were resuspended in 50 mM Tris–HCl (pH 7.5) buffer containing 150 mM NaCl. After five cycles of freezing (−80 °C) and thawing (37 °C), the phospholipid suspension was repeatedly extruded through a polycarbonate filter with the pore size of 100 nm (67). The resulting liposomes were stored at 4 °C and used within 1 week.

### PAP assay

PAP activity was measured at 30 °C for 20 min by following the release of water-soluble ^32^P_i_ from chloroform-soluble [^32^P]PA (1, 94). [^32^P]PA was enzymatically synthesized from 1, 2-dioleoyl DAG and [γ-^32^P]ATP with *E. coli* DAG kinase (94). The PAP reaction mixture contained 50 mM Tris–HCl (pH 7.5), 0.2 mM [^32^P]PA (5000–10,000 cpm/nmol), 2 mM Triton X-100, 1 mM MgCl_2_, 10 mM 2-mercaptoethanol, and the indicated amount of enzyme protein.

### Pah1–liposome interaction assay

Pah1 (0.1 μg) was incubated for 15 min at 30 °C with liposomes (10 mM phospholipid) in 50 mM Tris–HCl (pH 7.5) buffer containing 150 mM NaCl in a total volume of 20 μl. Following the incubation, the reaction mixture was centrifuged at 100,000*g* for 1 h at 4 °C, and the pellet was resuspended in the supernatant volume of the same buffer. The equal volumes of the supernatant and pellet fractions were subjected to SDS-PAGE (131), and the presence of Pah1 in the fraction was detected by immunoblotting (132, 133, 134) with rabbit anti-Pah1 antibody (59).

### Limited proteolysis

The purified preparation (0.1 μg) of Pah1 and its mutant derivatives was incubated at 23 °C with 25 ng of chymotrypsin in phosphate-buffered saline (pH 7.4) for various periods (0–16 min). The protein digests were separated by SDS-PAGE using 12% polyacrylamide gels and stained with SYPRO Ruby.

### Lipid analysis

[2-^14^C]acetate labeling of lipids (135), extraction (136), and separation by one-dimensional TLC (hexane/diethyl ether/glacial acetic acid [40:10:1 v/v]) on silica gel plates (137) were performed as described previously. After resolution, the ^14^C-labeled lipids were visualized by phosphorimaging with the Storm 860 Molecular Imager (GE Healthcare) and quantified by ImageQuant software (GE Healthcare) using a standard curve of [2-^14^C]acetate. To confirm the identity of the radiolabeled lipids, their migration on the silica gel was compared with authentic standards visualized by iodine vapor staining.

### Analyses of lipid droplets and nuclear/ER morphology

A Nikon Eclipse NiU microscope with an EGFP/FITC/Cy2/AlexaFluor 488 filter was used for the analysis of lipid droplets and nuclear/ER membrane morphology. Fields of view from one z-plane were captured with a DS-Qi2 camera, and images were analyzed with NIS-Elements BR software or FIJI software. Lipid droplets were imaged from stationary phase cells (absorbance at 600 nm ∼3.5) after staining with 2 mM BODIPY 493/503 in phosphate-buffered saline (pH 7.4) (27). Nuclear/ER membrane morphology was imaged from yeast cells expressing Sec63-GFP (33). The average number of lipid droplets per cell and percentage of cells with a round nucleus were scored from a total of ≥300 cells.

### SDS-PAGE and immunoblot analysis

SDS-PAGE (131) and immunoblotting (133, 134) were performed by standard procedures. Normalized gel loading was confirmed by protein concentration determination according to the method of Bradford (138) with bovine serum albumin as the standard. Protein transfer from polyacrylamide gels to polyvinylidene difluoride membranes was monitored by Ponceau S staining. The protein blots were probed with rabbit anti-Pah1 (2 μg/ml), rabbit anti-Cho1 (0.25 μg/ml), or mouse anti-Pgk1 (2 μg/ml) antibody. The antibody-treated membrane was incubated with alkaline phosphatase–conjugated goat anti-rabbit IgG antibody or goat anti-mouse IgG antibody at a dilution of 1:5,000, and the immune complexes were detected with an enhanced chemifluorescence substrate for alkaline phosphatase. The fluorescence signal from the immunoblot was detected by Invitrogen iBright 1500 imager, and the signal intensity was analyzed using the iBright Analysis Software.

### Data analysis

Kinetic parameters were determined by the enzyme kinetics module of the SigmaPlot software (Grafiti LLC). Microsoft Excel software was utilized for statistical analysis; *p* values <0.5 were taken to be statistically significant.

## Data availability

All data are contained within the article.

## Conflict of interest

The authors declare that they have no conflicts of interest with the contents of this article.
